# Employing Pneumatic, Telescopic Actuators for the Development of Soft and Hybrid Robotic Grippers

**DOI:** 10.3389/frobt.2020.601274

**Published:** 2020-11-11

**Authors:** Lucas Gerez, Che-Ming Chang, Minas Liarokapis

**Affiliations:** New Dexterity Research Team, Department of Mechanical Engineering, University of Auckland, Auckland, New Zealand

**Keywords:** hybrid actuation mechanism, pneumatic actuators, soft robotics, grasping, grippers and other end effectors, robotics

## Abstract

Traditionally, the robotic end-effectors that are employed in unstructured and dynamic environments are rigid and their operation requires sophisticated sensing elements and complicated control algorithms in order to handle and manipulate delicate and fragile objects. Over the last decade, considerable research effort has been put into the development of adaptive, under-actuated, soft robots that facilitate robust interactions with dynamic environments. In this paper, we present soft, retractable, pneumatically actuated, telescopic actuators that facilitate the efficient execution of stable grasps involving a plethora of everyday life objects. The efficiency of the proposed actuators is validated by employing them in two different soft and hybrid robotic grippers. The hybrid gripper uses three rigid fingers to accomplish the execution of all the tasks required by a traditional robotic gripper, while three inflatable, telescopic fingers provide soft interaction with objects. This synergistic combination of soft and rigid structures allows the gripper to cage/trap and firmly hold heavy and irregular objects. The second, simplistic and highly affordable robotic gripper employs just the telescopic actuators, exhibiting an adaptive behavior during the execution of stable grasps of fragile and delicate objects. The experiments demonstrate that both grippers can successfully and stably grasp a wide range of objects, being able to exert significantly high contact forces.

## 1. Introduction

Robotic end-effectors have evolved over the past few decades from simple, parallel jaw grippers to complex robot hands with multiple degrees of freedom (DoF) that require complex control laws and sophisticated sensing. Dexterous robot hands can efficiently grasp objects utilizing different types of actuation mechanisms, such as pulley and gear transmissions (Liu et al., 2008) or cable-driven systems (Xu and Todorov, 2016). In most cases, dexterous hands are rigid, difficult to develop, heavy, and hard to control (Kontoudis et al., 2015). Over the past decade, a series of adaptive end-effectors have been designed taking advantage of compliance and underactuation to achieve high grasping efficiency and robustness with simple and intuitive control. By introducing elastic elements into traditional robotic structures, grippers can successfully execute a variety of grasping tasks under object pose uncertainties (Kim et al., 2013) and with a plethora of objects (Liarokapis and Dollar, 2017). Such design approaches increase the area of the contact patches between the object and the end-effectors, distributing the grasping forces appropriately and enabling interaction with soft, deformable objects (Tai et al., 2016; Stuart et al., 2017).

More recently, following the path of intrinsical compliance in robotic devices, soft grippers based on completely soft pneumatic actuators (SPAs) have received an increased interest from the research community due to their flexibility, high customizability, and environmental adaptability (Sun et al., 2013). Rigid manipulators require a combination of joints and rigid links to execute grasps, while the motion of soft actuators is promoted by the intrinsic mechanical properties of soft materials. Soft robotic hands are suitable for grasping and manipulating delicate and/or fragile objects and complex shapes by conforming to the object's geometry and distributing contact forces among the surface (Galloway et al., 2016). This type of robot hands and grippers does not require specific control inputs, reducing the complexity, and cost of the device without compromising the grasping efficiency and safety (Laschi and Cianchetti, 2014). The high conformability of soft actuators can be an advantage when using soft grippers inside non-traditional environments (e.g., cavities and narrow spaces) or to grasp fragile objects. In unstructured and dynamic environments, the shape of the objects to be grasped cannot be predictable, so robot hands with high adaptability are desirable (Guarnieri et al., 2007).

Soft robotic grippers have been proven to be a good alternative when grasping different types of objects (Hao et al., 2016; Wei et al., 2016; Zhou et al., 2017; Li et al., 2018). Although the size of soft robotic grippers is a concern for robot gripper and hand designers, most of these devices have similar dimensions with the traditionally used bulky, rigid robot grippers. Another advantage of soft actuators is that they can be easily molded to shapes that reduce the amount of space taken when they are not inflated (e.g., accordion-style parts, origami structures, and foldable actuators) (Martinez et al., 2012; Paez et al., 2016; O'Neill et al., 2017; Gerez et al., 2020a).

**Figure 1:**
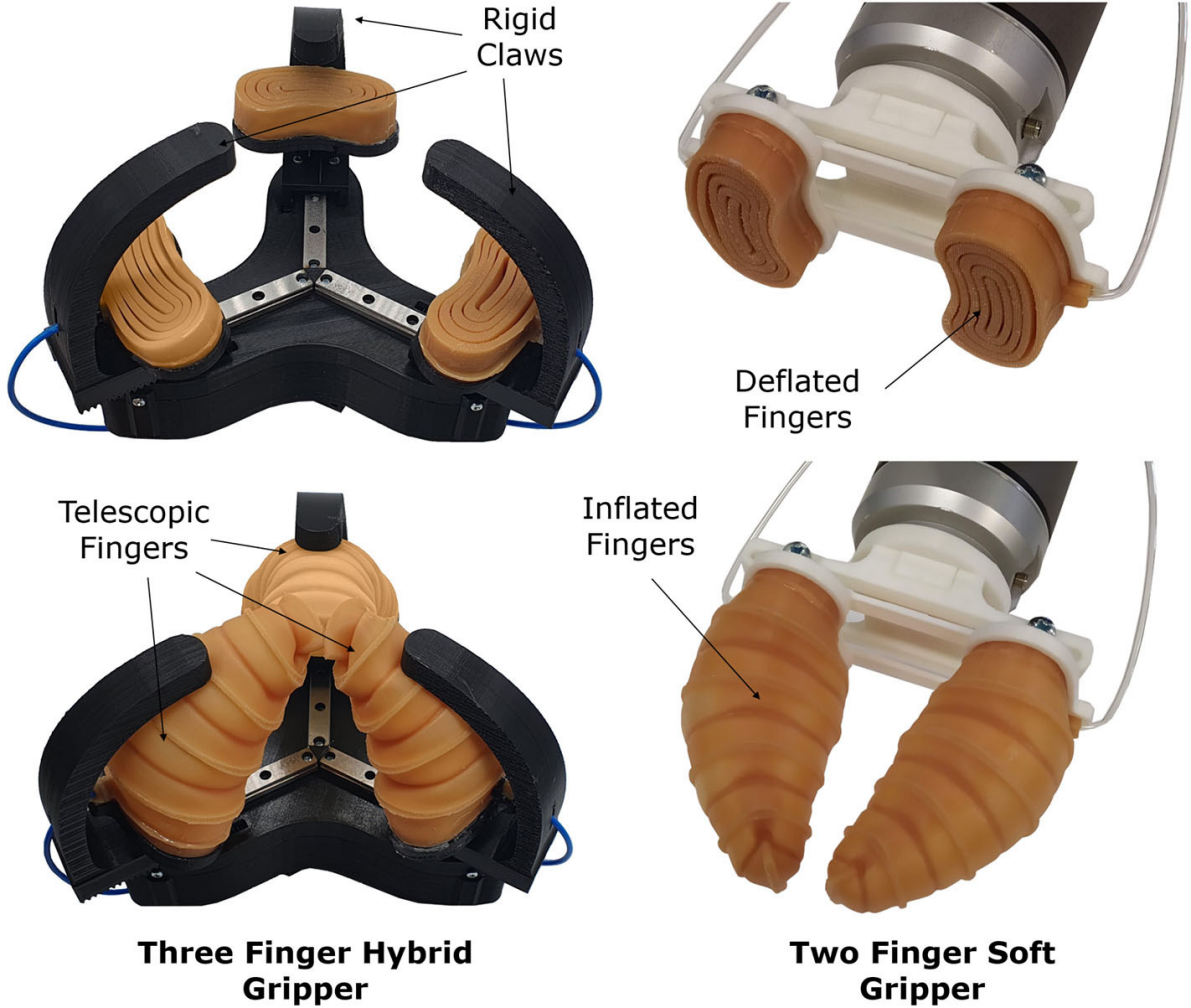
The hybrid robotic gripper **(left)** and the soft robotic gripper **(right)** that are equipped with the proposed retractable, telescopic, inflatable fingers. The robotic grippers are depicted in two different configurations: deflated (top) and inflated (bottom). The grippers can achieve stable grasps and can be used in unknown environments due to their high conformability and compactness.

In this paper, we propose soft, retractable, pneumatically actuated, telescopic actuators that can be used for the development of soft and hybrid robotic grippers (see Figure 1), facilitating the execution of efficient and stable grasps with a plethora of everyday life objects. The hybrid, encompassing, three-fingered robotic gripper combines curved rigid fingers/claws with pneumatically actuated telescopic mechanisms that improve grasp quality. The hybrid gripper combines the high forces exerted by rigid fingers with the adaptive behavior of soft actuators for executing a wide range of tasks. The soft gripper employs purely compliant fingers for stably grasping objects. The fingers consist of soft, pneumatic actuators that can be employed in scenarios that require an affordable (even disposable) device with minimal size. An example could be a gripper for handling of medical waste that can be easily stored. The performance of the proposed grippers is experimentally validated using three types of experiments: (i) grasping tests that involve different everyday objects, (ii) force exertion experiments that capture the maximum forces that can be applied by the fingers, and (iii) grasping disturbance resistance experiments that focus on calculating the amount of force that is required for an object to be released from the grasp.

The rest of the paper is organized as follows: section 2 discusses the related work, section 3 presents the design of the gripper, section 4 details the grasping experiments and the results, while section 5 discusses the findings and presents future directions.

## 2. Robot Grippers in Unstructured Environments

Robots and machinery used in unstructured and dynamic environments typically employ rigid, fully-actuated robotic grippers and hands. From autonomous search and rescue and fire fighting robots (Miyazawa, 2002; Hamins et al., 2015) to heavy machinery such as excavators (Sulaiman et al., 2015) or dual arm robot platforms used in earthquake recovery work (Egawa et al., 2013; Tadokoro et al., 2019), all these systems are equipped with different types of robotic end-effectors that employ rigid fingers. Such platforms are designed to manipulate irregular objects assisting humans in the execution of complex tasks. However, when working in the vicinity of humans in unstructured and complex environments, these systems require extensive human supervision for safety reasons. Notably, most of these machineries and platforms have exchangeable end-effectors, but only a few end-effectors have interchangeable fingers and appropriate contact surfaces for executing a wide range of applications. Modular exchangeable fingers are more commonly used in industrial grippers and applications. Examples include the Schunk range of grippers (SCHUNK GmbH, 2020) and the adaptive grippers of Festo (Festo Coorporate, 2020).

Regarding planning and control, traditional, rigid robot end-effectors typically require computationally expensive methods for performing versatile object manipulation and grasping (Ma et al., 2013). Alternatively, soft manipulators that employ soft pneumatic actuators combine actuation and structural components to enhance their adaptability and inherent compliance (Deimel and Brock, 2013; Zhou et al., 2017). Commercial designs, such as the Soft Robotics three-fingered soft gripper (Robotics, 2019) and the Festo tentacle gripper (Festo, 2017) are examples of soft grippers capable of grasping objects of various geometries by conforming to the object's surface. These types of pneumatic network actuators generally rely on expandable segments (Hao et al., 2016) to achieve complex motions, such as bending and twisting (Wang et al., 2018). The initial size of this type of actuators can be reduced by employing elastomeric origami structures (Martinez et al., 2012).

The human hand sits between rigid and soft manipulators in terms of precision, structural compliance, number of degrees of freedom and force exertion capabilities (Hughes et al., 2016). Similarly, designers of soft, adaptive robotic grippers also seek to obtain a balance between these characteristics. Following an extensive review of a plethora of robot hand designs that range from anthropomorphic robot hands (Gao et al., 2019; Piazza et al., 2019) to underactuated designs, such as the Yale OpenHand project devices (Ma et al., 2013) and the unconventional adaptive robot grippers and hands of New Dexterity (Chang et al., 2019), adaptive end-effectors employ soft materials and the benefits of structural compliance to increase grasp robustness. Structural compliance increases grasping robustness by maximizing the area of the contact patches between the gripper and the object. It also facilitates grasping under object pose uncertainties (Liarokapis and Dollar, 2017) and decreases the grasping force required to extract stable grasps (Majidi, 2014). Variable stiffness manipulators allow active change in gripper joint or contact surface compliance. This concept is commonly seen in soft manipulators where a pressure cavity changes the elastic behavior of the manipulator during actuation (Wei et al., 2016). Some other approaches feature laminar jamming structures (Gerez et al., 2020b) or other actuating methods, such as smart memory alloys (Wang and Ahn, 2017) and magnetorheological fluids (Pettersson et al., 2010). The variable stiffness capabilities allow grippers to interact with objects that would otherwise be hard to grasp but actively changing the structural compliance is control intensive and fabrication of the structures is more challenging than the fabrication of the concepts proposed in this work. Similarly, other hybrid grippers combine the compactness and high forces of tendon-driven systems and the compliance and conformability of soft actuators (Meng et al., 2020). Such grippers offer effective solutions for delicate grasping but do not increase the contact area and the grasping quality through the execution of caging grasps as the grippers proposed in this work.

Multi-fingered grippers often have the ability to utilize encompassing grasps to improve grasp robustness. Depending on the poses of the fingers and on the external forces acting on the grasped object, grasp stability is typically increased when the number of participating robotic fingers increases. Structural compliance of soft and adaptive multifingered grippers also increases grasp stability as it increases the ability of the device to conform to the object shape. This increases the grasp wrench space and the grasp robustness to external perturbations (Roa and Suárez, 2014). Due to the inherent increase in contact area, envelope grasps create spatial cradles that lock the object in position (Bélanger-Barrette, 2016).

## 3. Designs

In this section, we present the design of the telescopic, soft actuators and the hybrid, and soft robotic grippers, discussing their manufacturing process, functionalities, and operation.

### 3.1. Hybrid Gripper Design

**Figure 2:**
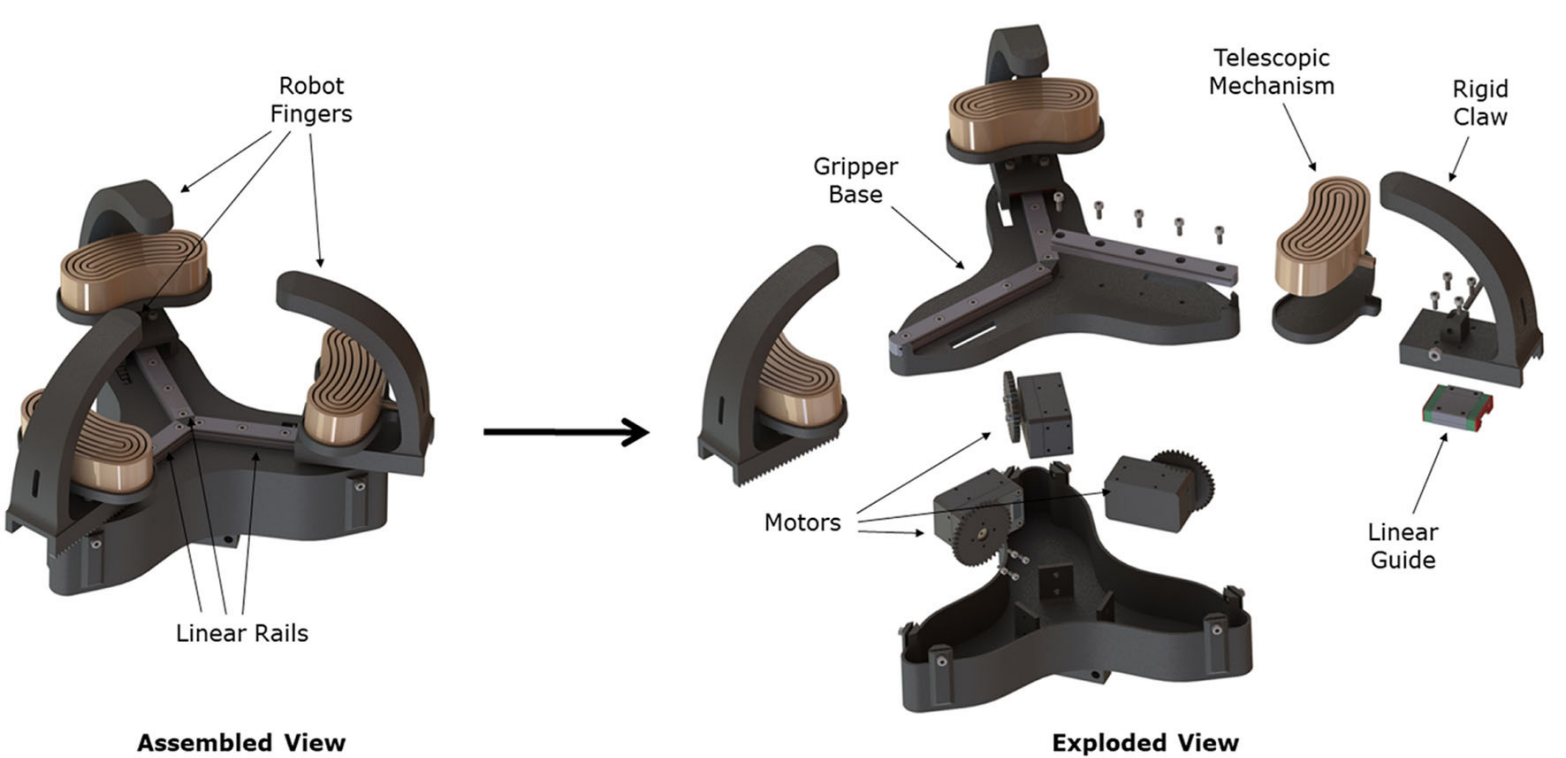
The hybrid, three-fingered robotic gripper consists of a gripper base, three rigid fingers/claws, and three soft, pneumatically actuated, telescopic mechanisms that act as soft fingers. Both finger types are attached on linear rails, which allow them to slide radially to the gripper center, closing the gripper and grasping the desired object. The fingers have a rack that is coupled to the motor gears for force transmission. Three Dynamixels XM430-W350 are used to move the fingers on the linear rails, while an external air supply is used to inflate the telescopic mechanisms. The soft fingers are mounted to the rigid claws using pin joints that allow them to follow the curvature of the rigid claws during inflation.

**Figure 3:**
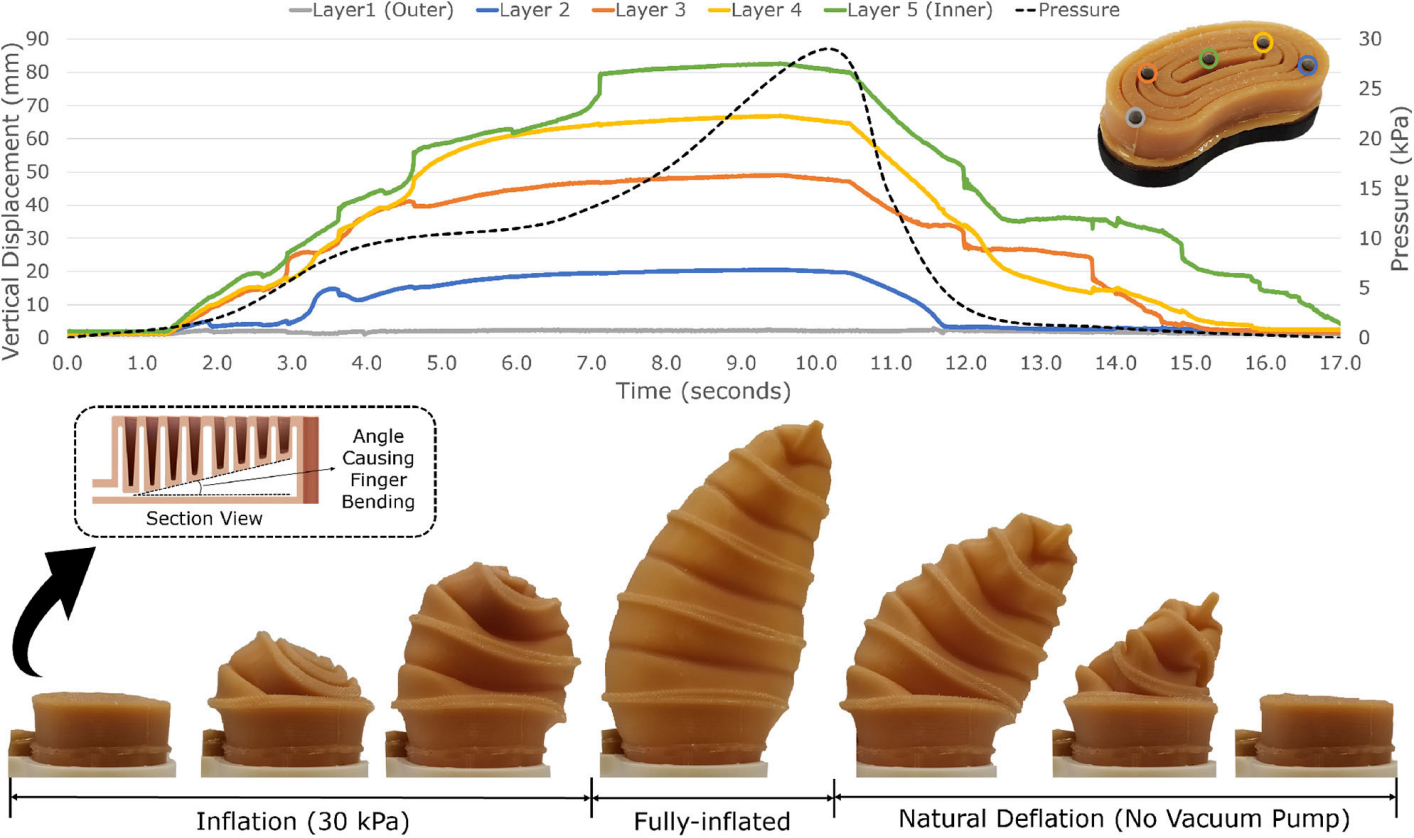
When the soft, telescopic actuator is inflated, each inner ring inflates separately, starting with the most exterior ring and finishing with the most interior one. The telescopic behavior allows object grasping with ease and stability. At the rest position, the soft actuator is 20 mm thick. However, when inflated, it can reach a height of more than 100 mm. The actuator can be inflated at different speeds, grasping objects quickly, or slowly. During the deflation process, the finger naturally returns to its original flat position without requiring any external forces or negative pressure inside the actuator (vacuum). Thus, retraction is completely passive. The finger bending is caused by an internal angle between the base of the actuator and the telescopic structure (see section view).

The proposed hybrid, robotic gripper consists of two main parts: the gripper base and three finger units (see Figure 2). Each finger unit consists of a 3D printed curved finger and an adjustable, angled, telescopic, pneumatically actuated, soft finger. The soft fingers are mounted on the base of the rigid claws through a spring-loaded pin joint. The pneumatic telescopic fingers are designed in a curved circular shape to facilitate bending during expansion (Figure 3). The telescopic mechanisms are made out of urethane rubber (Smooth-On Vytaflex 40) and operate at a pressure of 30 kPa. The soft fingers can conform to the geometry of various objects and appropriately distribute the contact forces allowing grasping of fragile and delicate objects. The finger units are mounted on three linear rails spaced equally around the center of the robotic gripper. The claws are 3D printed with PLA material, and driven via rack and gear couplings connected to three Robotics Dynamixel XM430-W350 smart actuators. The telescopic pads are connected to an external air supply line with 3 mm diameter polyurethane tubing, and the flow rate is controlled. The soft fingers can be actuated independently (when connected to individual air supplies) or synchronized (with a single compressed air supply).

The complete hybrid robotic gripper weighs 1,380 g, including the Dynamixel XM430-W350 motors (82 g each). The hybrid gripper has a fingertip aperture of approximately 104 mm in diameter when the fingers are fully opened and the claw height is 100 mm (distance between the base and the inner side of the fingertip). The clearance between the telescopic pads at rest (deflated) and the fingertip of the rigid claws is approximately 55 mm. The robot base was specifically designed so as to easily attach the robotic gripper to a robot arm (e.g., the UR10 robot arm of Universal Robots).

The proposed hybrid robotic gripper offers three different grasping modes: rigid grasping, soft grasping, and encompassing or envelope grasping. The rigid grasps are performed employing the curved, 3D printed claws by moving them radially. The claws can operate synchronously or independently controlling the different motor currents. Similarly, for soft grasping, the telescopic fingers motion can be controlled radially by the motors, but the contact area between the object and the gripper is exclusively established by the soft finger structures. During encompassing or envelope grasping, the object is caged by the gripper and pushed by the inflatable structures against the rigid claws so as to maximize the contact area between the object and the different parts of the gripper (both rigid and soft).

### 3.2. Soft Gripper Design

The soft robotic gripper consists of two main parts: a robot wrist/mount and two soft, inflatable, telescopic fingers. A control box is used to inflate and deflate the fingers. The control box contains a 12 V mini air pump and a solenoid valve. The air pump is in charge of inflating the fingers while the solenoid valve releases the pressure of the system so that the finger can retract to its original and flat position. The soft robotic fingers developed for this gripper are retractable and telescopic. More precisely, the fingers are flat when not inflated (20 mm long) and elongate to more than 100 mm long when inflated due to their telescopic structure (Figure 3). The fingers are called retractable because they naturally/passively return to their original flat position when the internal pressure is released, without requiring any external forces or negative pressure inside the actuator (if desired, vacuum can be applied to decrease the deflation time). The soft robotic finger can be inflated at different speeds (varying the flow rate in the inlet), being able to grasp objects either quickly and firmly or slowly, carefully, and delicately. The natural deflation of the actuator takes on average 7–8 s. The simplicity and the compactness of the proposed design lead to the development of cost-effective even disposable robotic grippers. Each inflatable finger costs less than 1 USD of material to be manufactured. Thus, the proposed soft robotic gripper is a good alternative for sterilized environments where the use of disposable products guarantees a high level of cleanness (e.g., dust-free factories and hospitals). In particular, in hospitals, such a gripper can be used to handle dangerous medical waste in a disposable manner to reduce the transmission of bacteria and viruses (e.g., COVID-19).

### 3.3. Soft Telescopic Actuator Design

The soft telescopic actuator is based on a urethane rubber (Smooth-On Vytaflex 40) structure designed for grasping assistance during the execution of activities of daily living. The foldable structure was designed in such a way that it does not influence the interaction with the environment when the gripper is not inflated due to its small thickness and telescopic behavior. The rounded shape of the actuator was chosen so as to increase aperture when deflated and therefore the size of the objects that could be caged when the structure gets inflated. The actuator operates at a maximum pressure of 30 kPa, weighs 50 g, is 20 mm thick, 90 mm wide, and the walls are 1 mm thick. The actuator's final dimensions were experimentally defined through several iterations to achieve the retractable behavior.

**Figure 4:**
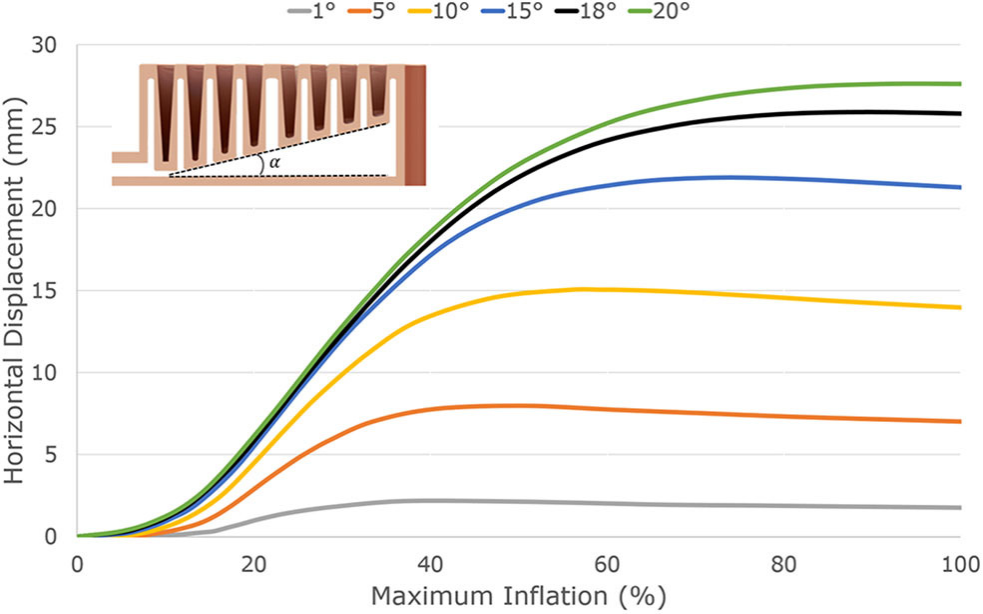
The finger bending is caused by an internal angle between the base of the actuator and the top of the actuator. Each telescopic ring has a different height, forcing the finger to extend unevenly when inflated, thus bending it. An FEM model of the cross-section of the soft actuator was developed in order to verify the effect of the internal angle on the horizontal displacement. In this analysis, all the internal walls were pressurized, while the base was constrained. The graph shows the horizontal displacement for the telescopic structure for different values of internal angle (α), ranging from 1deg to 20deg. The results demonstrate that the horizontal displacement of the actuator is higher when the internal angle of the actuator is increased. The telescopic actuator employed in the proposed grippers has an internal angle of 18deg. At internal angles higher than 18deg, the soft actuator partially loses its telescopic behavior and does not fully retract naturally/passively.

The finger bending is caused by an internal angle between the base of the actuator and the top of the actuator (see the section view in Figure 4). Each telescopic ring has a different height, forcing the finger to extend unevenly when inflated, thus bending it. A finite element method (FEM) model of the cross-section of the soft actuator was developed in order to verify the effect of the internal angle on the horizontal displacement. The proposed actuator was modeled employing the Mooney-Rivlin hyperelastic model (Ogden, 1997). Assuming that the urethane rubber is incompressible, the simulation used the following constants for the Mooney-Rivlin model: *C*_1_ = 76 kPa and *C*_2_ = 22 kPa. The FEM analysis was performed using ANSYS Workbench 2020 R1 computer-aided engineering (CAE) software. In this analysis, all the internal walls were pressurized while the base was constrained. Figure 4 shows the horizontal displacement for the telescopic structure for different values of internal angle (α), ranging from 1 to 20deg. The results demonstrate that the horizontal displacement of the actuator is higher when the internal angle of the actuator is increased. The telescopic actuator employed in the proposed grippers has an internal angle of 18deg. It was experimentally validated that at internal angles higher than 18deg, the soft actuator partially loses its telescopic behavior and does not fully retract naturally/passively.

**Figure 5:**
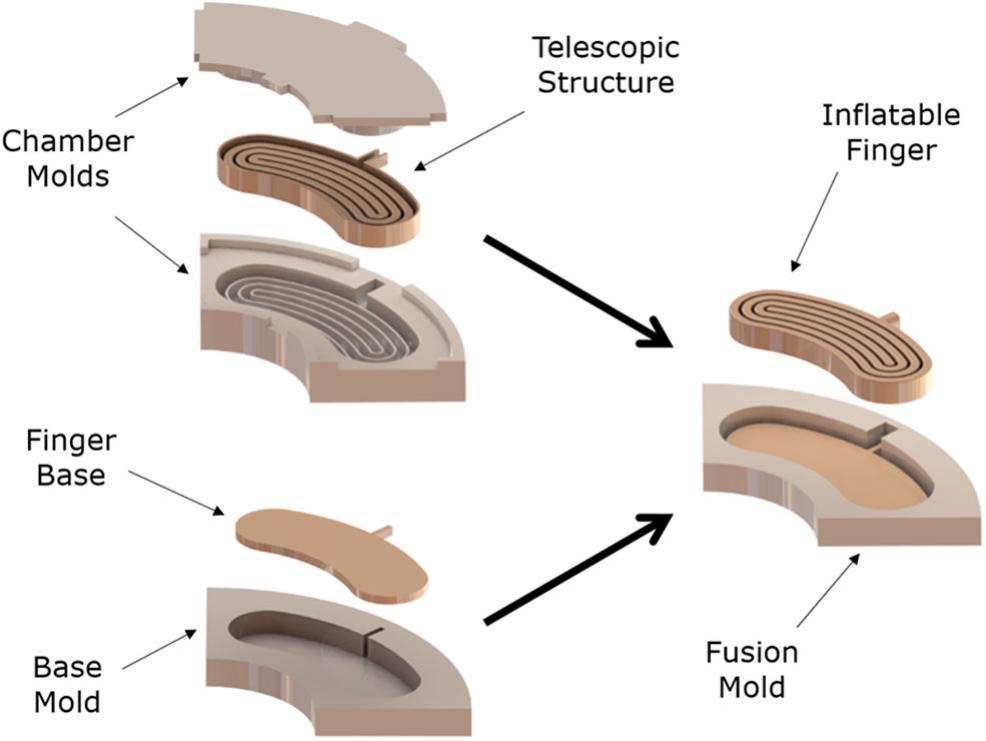
The manufacturing process of the inflatable, telescopic finger (made out of Smooth-On Vytaflex 40) involves the following three molding steps: (i) the foldable part of the actuator is fabricated using two separate molds, (ii) the base layer is fabricated using a third mold, with the base layer being 1.5 mm thick and 2 mm smaller than the foldable part in all directions so that they can be molded together, (iii) after both parts are cured, a forth mold is used to combine the upper part and the base layer part, filling the remaining gaps between the two parts and bonding them together. The final inflatable structure of the soft, telescopic mechanism is 20 mm thick and 90 mm wide.

The manufacturing process of the telescopic actuator involves three different molding steps. Figure 5 illustrates the steps that are required to manufacture the actuator. Initially, the foldable part and the base layer of the actuator are fabricated. The base layer is 2 mm smaller than the upper part in all directions so that they can be molded together. After both parts are cured, a third mold is used to combine the upper part and the base layer part, filling the remaining gaps between the two parts and bonding them together. This technique avoids leakages and deformations in the actuator. Although the actuator has a thick elastomer base, a fabric layer can also be added to the base to restrict the extension of the actuator along the base axes.

The actuator behavior during the inflation process can be seen in Figure 3. The motion of each layer was recorded using an optical motion capture system with eight cameras (Vicon Motion System Ltd., UK). The tracking markers were added to each layer as shown in Figure 3. When the soft telescopic actuator is inflated, each inner ring inflates separately, starting with the most exterior ring and finishing with the most interior one. The telescopic behavior allows object grasping with ease and stability. During its rest position, the soft actuator is 20 mm thick. However, when inflated, it can reach more than 100 mm of height, having an elongation ratio bigger than five (height of the actuator in the inflated state divided by the height of the actuator in the rest state). When compared to the state-of-the-art of soft, telescopic actuators, the proposed telescopic actuator has the highest elongation ratio among all the examined solutions. Table 1 shows the elongation ratio of different soft, telescopic actuator designs found in the literature.

**Table 1:** Comparison of the elongation ratio of multiple soft actuators (height of the actuator in the inflated state, *L*_*i*_, divided by the height of the actuator in the rest state, *L*_*r*_).

**Soft actuator**	***L*_*i*_/*L*_*r*_**
Connolly et al. (2015)	1.2
Bryant et al. (2014)	1.4
Deshpande et al. (2017)	2.1
Li et al. (2017)	1.9
Martinez et al. (2012)	3.6
Rafsanjani et al. (2018)	1.25
Soft telescopic actuator (this study)	5.3

*It is evident that the proposed actuator outperforms previously proposed soft actuators in terms of elongation performance*.

## 4. Experiments and Results

The experiments that were conducted to assess the performance of the robotic grippers were divided into three parts. The first part focused on evaluating the use of the grippers for grasping a plethora of everyday life objects. The second experiment measured the forces that the devices are capable to exert. The third experiment focused on assessing the efficiency of the grippers on resisting external forces that try to pull the objects away from the grasp evaluating grasping disturbance resistance.

### 4.1. Grasping Experiments Involving Everyday Life Objects

The first experiment was executed in order to evaluate the grasping performance of the grippers for various everyday life objects. The goal of these tests is to verify if the proposed grippers are capable of executing different grasping tasks. A total of 13 objects were tested. The objects were selected from the YCB object set (Calli et al., 2015), an object set designed for facilitating benchmarking in robotic manipulation and grasping. The objects ranged from a tiny die to a heavy hammer (the objects' dimensions and weights can be found in Table 2). Each object was placed on a flat surface, and the gripper was connected to the robot arm. The grasping position and postures were determined through manual kinesthetic teaching and teaching by demonstration using the gravity compensation mode of the robot arm. Five grasping attempts were executed for each object. After executing the grasp, the robot arm lifts the object and holds it for 10 s. The robot gripper is then moved repeatedly in the horizontal direction and finally placed back on the surface. A grasp is considered as both successful and stable if no visible object reorientation (motion in any direction) or slippage occur during the task execution.

**Table 2:** Grasp stability results.

**YCB object**	**Properties**		**Soft gripper**		**Hybrid gripper**					
	**Weight** **(g)**	**Dimensions** **(mm)**	**(Soft grasp)**		**(Rigid grasp)**		**(Soft grasp)**		**(Encompassing)**	
			**Grasp**	**Stability**	**Grasp**	**Stability**	**Grasp**	**Stability**	**Grasp**	**Stability**
Master chef can	414	102 × 139	Y	Y	Y	Y	Y	Y	Y	Y
Mustard bottle	431	50 × 85 × 175	Y	Y	Y	Y	Y	Y	Y	Y
Fork	34	14 × 20 × 198	Y	Y	Y	Y	Y	Y	Y	Y
Plastic wine cup	133	89 × 137	Y	Y	Y	Y	Y	Y	Y	Y
Jelly box	97	28 × 85 × 73	Y	Y	Y	Y	Y	Y	Y	Y
Wooden cube	378	90 × 90 × 90	Y	Y	Y	Y	Y	Y	Y	Y
Plastic banana	66	36 × 190	Y	Y	Y	Y	Y	Y	Y	Y
Tennis ball	58	64.7	Y	Y	Y	Y	Y	Y	Y	Y
Marble	5.3	16	Y	Y	Y	Y	Y	Y	Y	Y
Dice	5.2	16.2	Y	Y	Y	Y	Y	Y	Y	Y
Chain	100	2 × 4 × 130	Y	Y	Y	Y	Y	Y	Y	Y
Hammer	688	32 × 40 × 160	N	N	Y	Y	Y	N	Y	Y
Pan	950	270 × 25 × 30	N	N	N	N	N	N	Y	Y

*The different grasping modes (Soft, Rigid, and Encompassing) of the proposed robotic grippers are evaluated. Stability is evaluated as the ability of the gripper to retain a stable grasp during the execution of an arm trajectory that introduces significant disturbances (as seen in the Supplementary Video 1)*.

**Figure 6:**
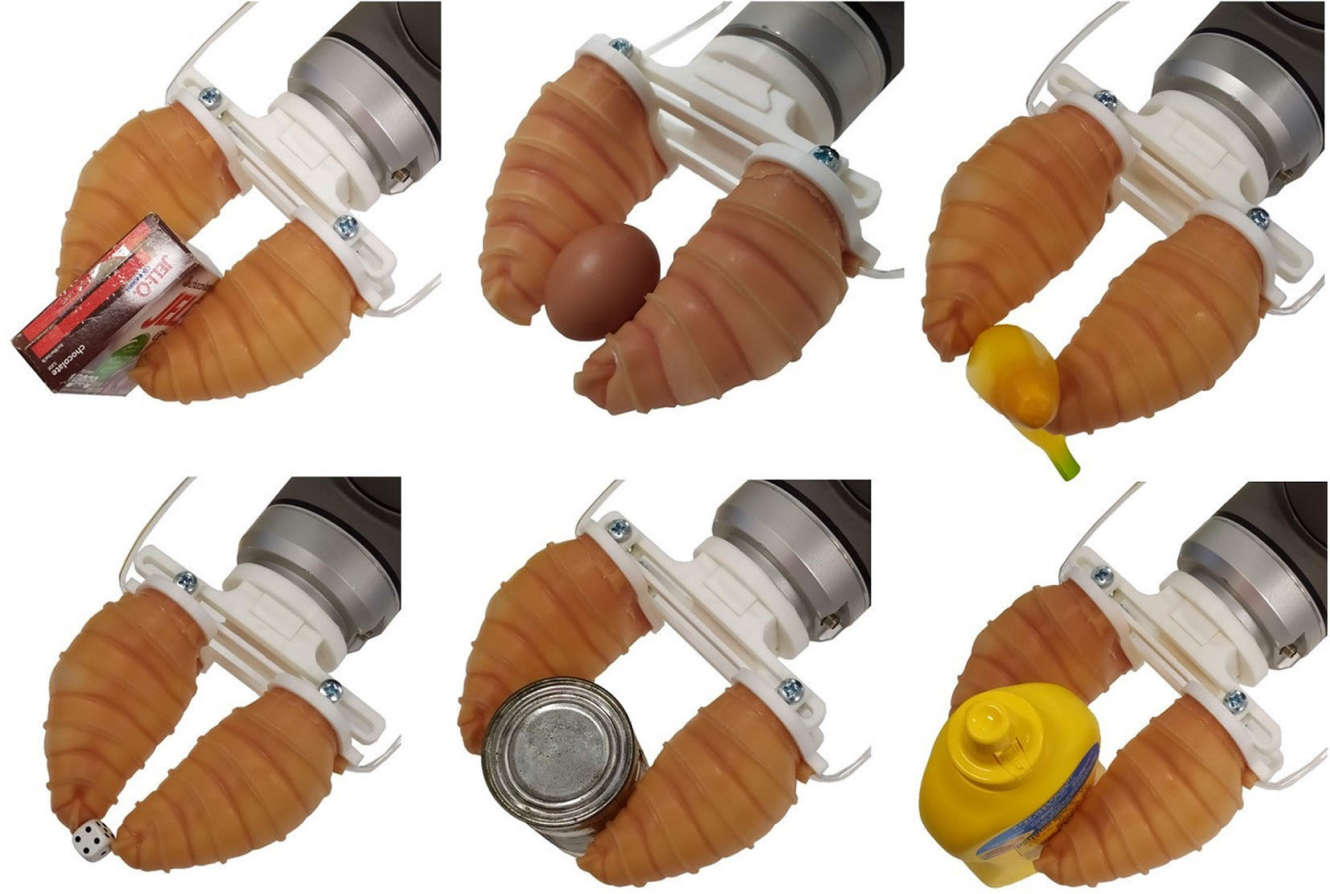
Grasping experiments were conducted to evaluate the performance of the soft robotic gripper for grasping different everyday life objects. Each object was placed on a flat surface and the gripper was attached on a Universal Robots UR10 robot arm. Upon grasp execution, the robot arm was lifting the object and was holding it steady for 10 s. The robot gripper was then moved in the horizontal direction and finally was placed back on the surface. A successful grasp was also considered as stable if there was no visible object reorientation (motion in any direction) or slippage during task execution. A total of 11 out of the 13 objects examined were both successfully and stably grasped.

A total of 11 out of 13 objects were successfully grasped (see Figure 6) by the soft gripper, while the hammer and the pan could not be grasped (see Table 2). During the experiments, it was noticed that grasps with high contact area between the object and the gripper were easily executed. The hammer and the pan were the heaviest objects among all tested objects. The contact area between these objects and the soft gripper was significantly small, hindering a successful and stable grasp.

**Figure 7:**
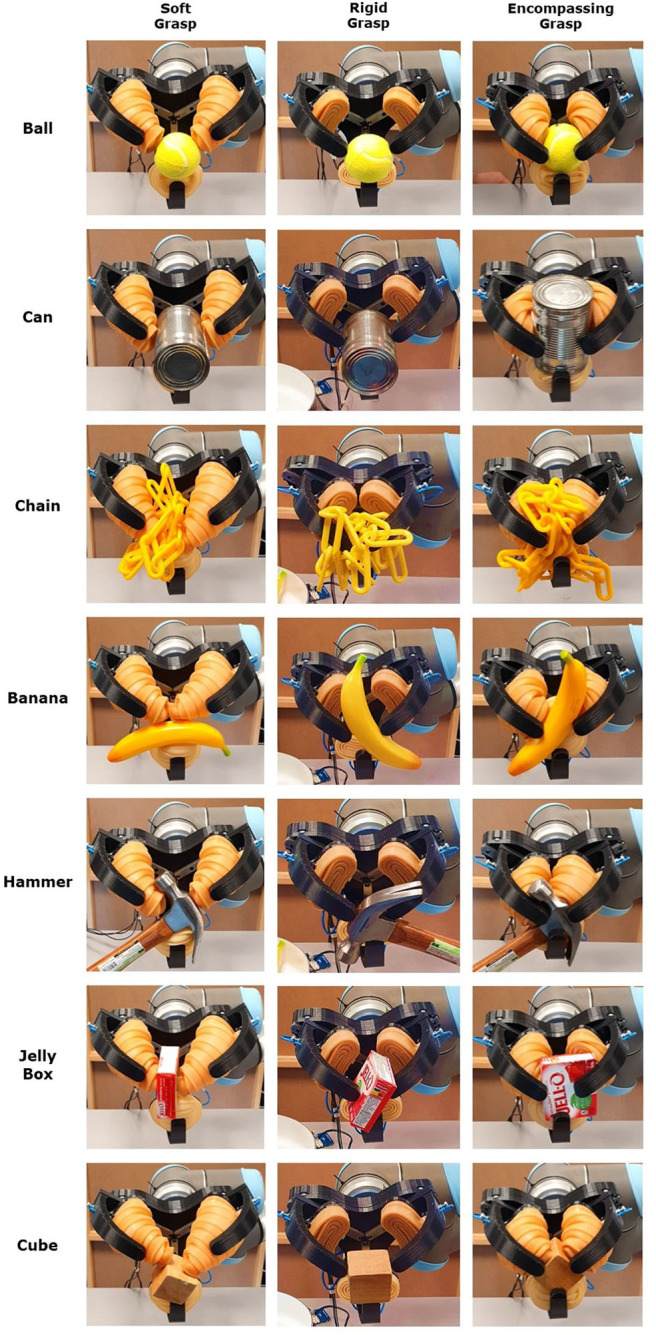
The three-fingered, hybrid robotic gripper offers three different grasping modes: rigid grasping, soft grasping, and encompassing (or envelope/caging) grasping. This figure presents successful grasps achieved for all modes for seven everyday life objects: a ball, a can, a chain, a banana, a hammer, a jelly box, and a cube.

The experiments conducted with the hybrid robotic gripper employed all three different grasping modes (rigid grasping, soft grasping, and encompassing grasping). The experimental evaluation of encompassing grasping capabilities was performed with the same procedure as grasping with pinch grasps (rigid grasping). However, in order to allow successful caging, objects were elevated from the table surface. The results are presented in Table 2 and Figure 7, showing that the gripper was able to grasp all twelve objects, however, the pan and the hammer could not be stably grasped using all three operation modes of the gripper. Due to the compliance of the soft fingers, the gripper cannot grasp heavy objects (such as the hammer and the pan) in the soft grasping mode. On the other hand, the encompassing grasping mode was proved to be efficient in grasping heavy objects with small contact area, due to the caging capabilities.

### 4.2. Grasping Force Experiments

**Figure 8:**
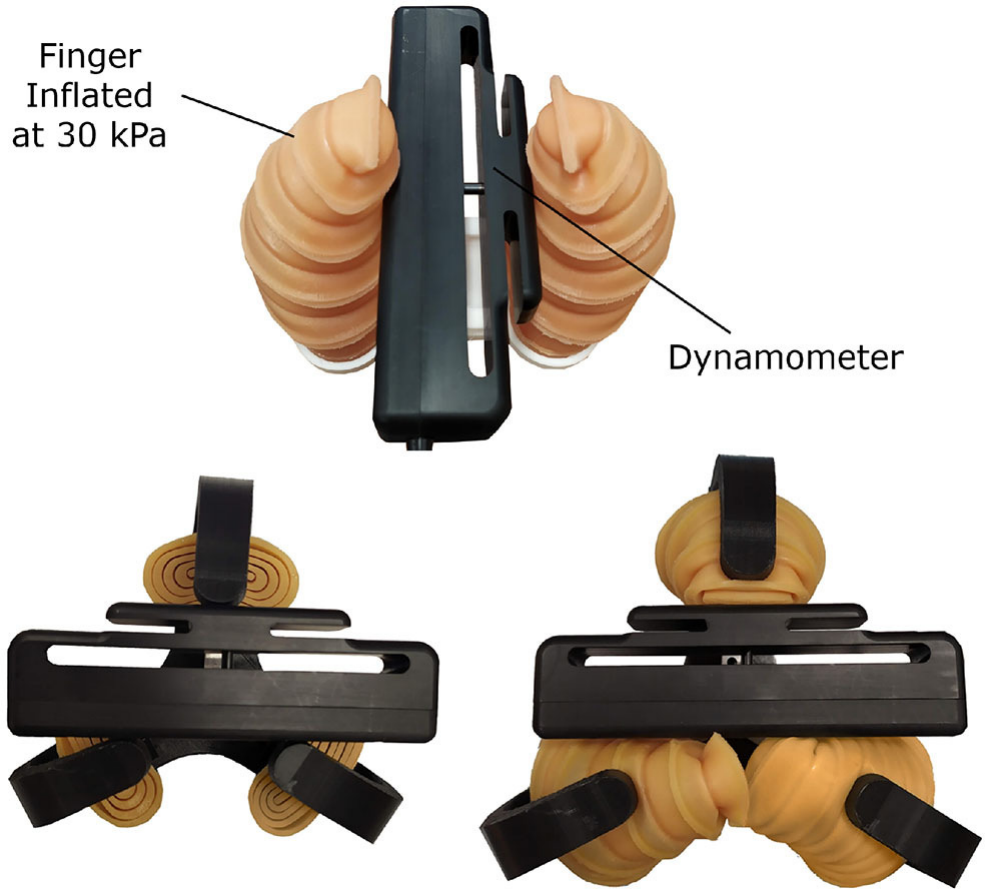
The force exertion capabilities of the soft and hybrid robotic grippers were experimentally evaluated using a dynamometer. During the experiment, the dynamometer was used to measure the forces exerted by the robotic fingers while grasping the device. The dynamometer was placed in multiple positions between the fingers and the maximum possible contact force was exerted. For the soft robotic gripper the maximum force obtained was 8.1 N **(top)**, while for the hybrid robotic gripper the maximum force exerted was 59.9 N for the rigid grasp and 12.8 N for the soft grasp **(bottom)**.

The second experiment focused on measuring the contact forces that the devices can apply while grasping objects. In this experiment, a Biopac MP36 data acquisition unit (Biopac Systems, Inc., USA) was used with the SS25LA dynamometer to measure the forces exerted by the robotic grippers, as shown in Figure 8.

During the experiments with the soft robotic gripper, the system was actuated until the maximum pressure of the actuator was reached (30 kPa). The dynamometer was placed in different positions between the fingers. The maximum force obtained during the experiments was 8.1 N. The maximum force was reached close to the base of the actuator (stiffer region compared to the tip of the soft finger). According to Polygerinos et al. (2015), the required force to grasp objects during activities of daily living does not exceed 10–15 N. Although the amount of force applied by the fingers is not considered high when compared to rigid grippers (Kontoudis et al., 2015; Ma and Dollar, 2017), the large contact region and high friction between the object and the soft material of the finger compensate the lower forces, and the gripper is able to grasp a large range of objects. Also, the amount of force applied by the proposed soft robotic gripper is within the range of forces that the particular class of grippers can usually achieve (Sun et al., 2013; Wei et al., 2016; Li et al., 2018). The gripper can be improved to exert higher forces by increasing the thickness of the walls or changing the finger material. Stiffer walls of the actuator would increase the maximum pressure that the actuator can withstand and, consequently, increase the amount of force that the soft robotic gripper can apply.

During the experiments conducted with the hybrid robotic gripper, the dynamometer was placed horizontally between the fingertips so as for the two fingers to support one side of the dynamometer and the remaining third finger to support the opposite, measuring face of the dynamometer as shown in Figure 8. The gripper was then actuated with all three fingers simultaneously until motor stall was reached. The contact forces of the inflated soft padding were measured by placing the dynamometer horizontally in multiple positions between the fingers. The fingers were locked in their maximum position, and the telescopic fingers were inflated until maximum pressure was reached, as shown in Figure 8. The results are presented in Table 3. The gripper can exert almost 60 N of clench force. The hybrid gripper motors increase the forces exerted, resulting in clench forces 6+ times higher than the forces exerted by the soft gripper.

**Table 3:** Experimental evaluation of the force exertion capabilities of the proposed soft and hybrid robotic grippers.

**Robot gripper**	**Grasp type**	**Maximum clench force**
Soft gripper	Soft grasp	8.1 N
Hybrid gripper	Soft grasp	12.8 N
	Rigid grasp	59.9 N

*Both grippers are able to exert significant contact forces for the different grasping modes*.

### 4.3. Grasping Disturbance Resistance Experiments

**Figure 9:**
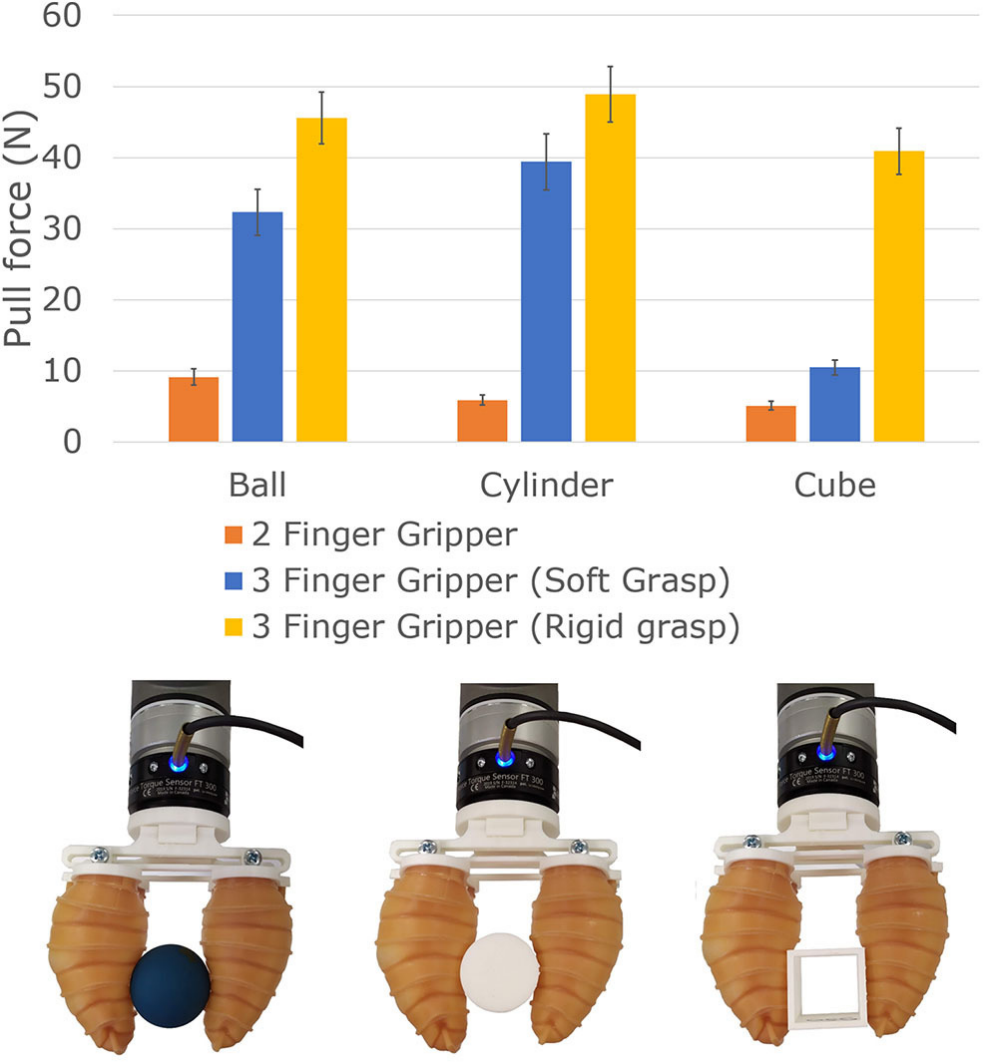
The amount of force required to pull the objects out of the grasp for the two robotic grippers was also measured. Three different types of objects were used: a sphere, a cube, and a cylinder. For the soft robotic gripper, the force required to pull the ball away from the gripper exceeded 10 N, while for the cylinder and the cube these forces were between 5 and 6 N. For the hybrid robotic gripper, the force required to pull the objects away from the gripper exceeded 40 N.

The third experiment focused on measuring the amount of force required to pull objects away from the grasps achieved by the robotic grippers. In this experiment, three different types of objects were used: a rubber sphere (41 mm), a PLA plastic cube (70 × 70 × 70 mm), and a PLA plastic cylinder (50 × 50 mm). The telescopic gripper was mounted on a robot arm (Universal Robots UR10) equipped with a force/torque sensor (FT300, Robotiq, Canada), and each object was grasped by the grippers with maximum force (at stalling torque for the motors and at 30 kPa of pressure for the telescopic fingers). The object was then pulled off the grippers, and the maximum force required for each trial was recorded. The experiment was repeated five times for each object. The object was pulled at different speeds to simulate different environmental conditions. The experimental results are reported in Figure 9.

For the soft robotic gripper, the force required to pull the ball away from the gripper reached more than 10 N, while for the cylinder and the cube, the forces were mostly between 5 and 6 N. The ball is made out of rubber, which increases the friction between the object and the gripper. The results demonstrate that the gripper can resist the same range of pulling forces as the soft robotic grippers found in the literature (Hao et al., 2016; Li et al., 2018). It was noticed that the resistance force between the object and the gripper could be increased by having a larger bending angle of the fingers. Such change would cage the object, creating a higher resistance against external forces. The bending angle of the finger can be changed by increasing the internal angle of the actuator (described in the section view of Figure 3).

For the hybrid robotic gripper, the objects were grasped by the claws at stalling torque and similarly by the telescopic pads at a pressure of 30 kPa. The object was then pulled off the gripper, and the maximum force required for each trial was recorded. The object was pulled at different speeds to simulate different environmental conditions. The mean maximum values of the experimental results is reported in Figure 9. The results demonstrate that the gripper can resist a large range of grasp disturbances achieved through the exertion of object pulling forces. It can also be noted that the hybrid gripper offers high resisting forces (in the soft and rigid grasping modes) for the ball and the cylinder, while the pull-off forces for the cube are much higher for rigid grasps than for soft grasps. This happens because the cube is not caged in the soft structure, and at least one of the edges of the cube is in direct contact with the telescopic actuators, reducing the contact area between the object and the gripper.

### 4.4. Video Demonstration

A video presenting the experimental validation of the efficiency of the proposed robotic grippers and demonstrating some of their uses, can be found at the following URL:

https://youtu.be/kBHql-gJcEI.

## 5. Discussion and Future Directions

In this paper, we presented soft, retractable, pneumatically actuated, telescopic actuators that facilitate the development of soft and hybrid robotic grippers, allowing for the efficient and stable execution of grasps involving a wide range of objects. Two different robotic grippers that employ the proposed telescopic mechanisms were developed. The soft robotic gripper is equipped with retractable, telescopic fingers that offer increased grasp stability, grasping of delicate, and fragile objects, as well as grasping of a wide range of everyday life objects. The experiments demonstrated that the gripper can efficiently grasp most of the examined daily life objects exerting a significant amount of force despite its non-traditional behavior. The soft robotic gripper is low-cost and the telescopic structure can be used in a disposable manner, facilitating the execution of specialized tasks that require a single use (e.g., grasping in sterilized environments, handling of medical waste, etc.). The hybrid robotic gripper combines three rigid claws that can exert significant contact forces with pneumatically actuated, soft telescopic mechanisms, offering three grasping modes: rigid grasping, soft grasping, and encompasing, caging grasping. The synergistic combination of the rigid and elastic parts allows the gripper to combine the advantages of traditional rigid robotic grippers (significant force exertion capabilities) with the advantages of the soft robotic grippers (delicate grasping of fragile objects), as well as to increase the overall grasping efficiency through the encompassing strategies. The experiments have demonstrated that the encompassing grasping mode is effective when grasping heavy objects that have a center of mass outside the grasping region (such as the pan demonstrated in the Supplementary Video 1). These types of objects are hardly grasped by fully soft or fully rigid grippers.

Regarding future improvements, the soft telescopic pads can be further improved so as to exert higher forces by increasing the thickness of the walls or changing the finger material. Stiffer walls of the actuator will increase the maximum pressure that the actuator can withstand and, consequently, increase the amount of force that the soft robotic gripper can apply. This scalability is desirable when developing hybrid systems of variant size and functionality for applications with different requirements. We also plan to integrate force sensors into the soft structure of the grippers so as to control the forces that are being applied by the actuators.

## Data Availability Statement

The original contributions presented in the study are included in the article/Supplementary Material, further inquiries can be directed to the corresponding author/s.

## Author Contributions

LG contributed to the ideas and designed and manufactured the robot grippers. LG and C-MC executed the proposed experiments. ML contributed to the ideas and supervised the project. All authors prepared the manuscript collectively.

## Conflict of Interest

The authors declare that the research was conducted in the absence of any commercial or financial relationships that could be construed as a potential conflict of interest.
